# Comprehensive Screening and Validation of Stable Internal Reference Genes for Accurate qRT-PCR Analysis in *Holotrichia parallela* under Diverse Biological Conditions and Environmental Stresses

**DOI:** 10.3390/insects15090661

**Published:** 2024-08-30

**Authors:** Zhongjun Gong, Jing Zhang, Qi Chen, Huiling Li, Ziqi Zhang, Yun Duan, Yueli Jiang, Tong Li, Jin Miao, Yuqing Wu

**Affiliations:** 1 Henan Key Laboratory of Crop Pest Control, Key Laboratory of Integrated Pest Management on Crops in Southern Region of North China, Institute of Plant Protection, Henan Academy of Agricultural Sciences, Zhengzhou 450002, China; gongzj_2@hotmail.com (Z.G.);; 2 Luohe Academy of Agricultural Sciences, Luohe 462300, China; 3 Institute of Plant Protection, Luoyang Academy of Agricultural and Forestry Sciences, Luoyang 471027, China

**Keywords:** *Holotrichia parallela*, qRT-PCR, reference gene screening, stability validation

## Abstract

**Simple Summary:**

The dark black chafer, *Holotrichia parallela* Motschulsky (Coleoptera: Scarabaeidae), is an important subterranean insect in China due to its wide distribution and the high degree of damage it causes. Selecting stable reference genes is crucial for accurate quantitative polymerase chain reaction (qPCR) and gene expression analysis. This study evaluated the expression stability of 11 candidate reference genes in *H. parallela* under various biological conditions and environmental stresses. Our findings suggest that the optimum reference genes were as follows: *RPL18* and *RPL13a* for developmental stages and RNAi conditions, *RPL13a*, *RPL18*, and *RPL32* for female and male adults, *RPL13a* and *RPS3* for different tissues, *RPL32*, *RPL13a*, and *RPS3* for varying photoperiod conditions, and *Actin* and *RPL13a* for different temperatures. These discoveries will serve as a foundation for subsequent precise qPCR and gene expression studies in *H. parallela* and other closely related insect species.

**Abstract:**

*Holotrichia parallela* is among the world’s most destructive pests. For accurate qPCR and gene expression studies, the selection of stable and appropriate reference genes is crucial. However, a thorough evaluation of potential reference genes for use in *H. parallela* research is lacking. In this study, 11 reference genes (*GAPDH*, *RPL32*, *RPL7A*, *RPS18*, *RPL13a*, *RPL18*, *Actin*, *RPS7*, *RPS3*, *VATB*,and *EF1A*) were evaluated under different biological conditions and environmental stresses. The stability of 11 potential reference gene transcripts was evaluated through various computational tools, including geNorm, BestKeeper, NormFinder, theΔCt method, and the RefFinder program. Under various developmental stages and RNAi conditions, *RPL18* and *RPL13a* exhibited the greatest stability. *RPL13a*, *RPL18*, and *RPL32* were the most stable genes in both male and female adults. Under differing tissue conditions, *RPL13a* and *RPS3* stood out as the most reliable. Moreover, under varying photoperiod conditions, *RPL13a*, *RPS3* and *RPL32* were the most stable genes. Lastly, *Actin* and *RPL13a* were the most stable genes across different temperatures. These findings offer essential criteria for selecting suitable reference genes across diverse experimental settings, thereby establishing a solid basis for accurate gene expression studies in *H. parallela* using RT-qPCR.

## 1. Introduction

The reverse transcriptase-quantitative polymerase chain reaction (RT-qPCR) is an efficient, dependable, and reproducible technique that facilitates the accurate measurement of gene expression during various biological processes [1]. A widely adopted strategy in RT-qPCR for normalizing gene expression data involves the concurrent measurement of the expression of an reference gene within the same sample, ensuring the accuracy and reliability of the results [1]. However, it is important to note that the levels of expression for frequently used reference genes may exhibit considerable variation across diverse experimental settings [2,3,4,5,6]. Therefore, it is advisable to conduct a thorough and species-specific investigation to ascertain appropriate reference genes [3,7].

*Holotrichia parallela* is an important subterranean pest in China because of its wide distribution and the significant damage it causes, which is usually between 20 and 30%. In severe cases, losses may exceed 50% [8,9,10]. The larvae, referred to as white grubs, dwell underground and consume the roots of crops [11]. This makes the larvae susceptible to infection by pathogens present in the soil [12].

In recent times, the adoption of agricultural practices, such as no-till and reduced tillage methods, along with straw return, has fostered favorable conditions for the survival and proliferation of white grubs, resulting in a significant increase in the *H. parallela* population [13]. Although chemical insecticides have been widely utilized to effectively control white grub infestations, this approach has unfortunately resulted in soil and groundwater contamination, leading to significant environmental issues [14]. Furthermore, the indiscriminate and improper utilization of chemical pesticides has fostered the emergence of resistance in grubs, thereby contributing to the progressive intensification of pest infestations in certain regions on an annual basis [12].

The advent of RNA interference (RNAi) technology has revolutionized the field of functional genomics, emerging as a potent tool for deciphering gene function and validating genetic targets [15,16]. RNAi serves as a pivotal strategy in agricultural plant protection [17]. Gene expression analyses are crucial for understanding the molecular mechanisms of physiological, developmental and reproductive processesin coleopteran insects, particularly given the high effectiveness and systemic penetration of RNAi in these species [15]. To date, these research efforts have successfully identified several genes in *H. parallela* [18,19,20,21]. However, to further our understanding and ultimately develop effective control strategies for this pest, it is imperative to precisely ascertain the gene expression levels of this pest under diverse biological conditions and environmental stresses.

The aim of this study was to establish a set of stably expressed reference genes in *H. parallela* for RT-qPCR analysis under various conditions, including different developmental stages, tissues, sexes, temperature treatments, light treatments, and RNAi treatments. To accomplish this, 11 potential reference genes were selected from the transcriptomes of *H. parallela*. These genes were glyceraldehyde 3-phosphate dehydrogenase (*GAPDH*), Vacuolar-type ATPase B (*VATB*), actin (*Actin*), elongation factor 1-alpha (*EF1A*), and ribosomal proteins (*L7a*, *L13a*, *L18*, *L32*, *S3*, *S7*, and *S18*). Many of these genes encode for proteins involved in essential cellular processes, such as glycolysis (*GAPDH*), energy transduction (*VATB*), cytoskeleton organization (*Actin*), protein synthesis (*EF1A*), and ribosome assembly (ribosomal proteins). All these genes are commonly used reference genes and were previously examined across other insect species [22,23,24]. The stability of these genes was then evaluated using four commonly used algorithms, namely BestKeeper, NormFinder, geNorm, and comparative ΔCt, as well as a comprehensive RefFinder program. These findings will enhance gene expression studies and facilitate gene function research in *H. parallela*, benefiting future investigations.

## 2. Materials and Methods

### 2.1. Experimental Materials and Treatment Methods

The *H. parallela* specimens utilized in the study were captured under a black light or collected from elm trees near the Henan Research and Development Center for Modern Agriculture, located in Yuanyang County, Henan, China (35°00′ N, 113°40′ E). Laboratory-reared *H. parallela* beetles were maintained under controlled conditions of 25 °C ± 1 °C temperature, 70% humidity, and a 16-hour light/8-hour dark photoperiod [25]. Samples were collected and dissected in accordance with rigorous experimental guidelines. The study examined the developmental stages of *H. parallela*, ranging from eggs to third-instar larvae. Adult tissues, including the head, prothorax, legs, and fat body, were dissected. Sex-specific samples, including the head and ovary or testis, were dissected for female and male adults, respectively.

The female beetles, comprising a total of 5 groups with 20 individuals in each, were placed in culture boxes with dimensions of 16 × 24 cm. Each culture box contained a layer of 30 cm of sterile soil. For the photoperiod experiments, adults were treated under five different light conditions (24L:0D, 16L:8D, 12L:12D, 8L:16D, 0L:24D) for four days [26]. Randomly, three beetles were chosen from within each group and dissected to isolate their heads.

Third-instar larvae were selected for temperature effect assessment [27] and cleaned before being subjected to treatments at 4 and 30 °C for 2 h each, with consistent humidity and light conditions between treatments.

Female beetles were fed elm leaves soaked in RNAi solutions (ddH_2_O, dsGFP, and dsFcp3C) for 11 days, as documented in prior research [20]. The ovaries of female beetles from each treatment were randomly selected, dissected, and transferred into sterile 1.5 mL centrifuge tubes.

The tissue dissection method for *H. parallela* was as follows. The samples to be dissected were fixed on paraffin dishes with clean insect needles and observed under a stereomicroscope. Chilled 1×PBS buffer solution was poured into the dish, and the tissues required for the experiment were dissected using dissecting forceps according to experimental requirements. Three biological replicates per sample group were frozen in liquid nitrogen and stored in 1.5 mL centrifuge tubes at −70 °C for future use.

### 2.2. RNA Isolation and Reverse Transcription

Total RNA was isolated from each sample using Trizol reagent (Invitrogen, Carlsbad, CA, USA) following the manufacturer’s instructions. RNA purity and concentration were analyzed spectrophotometrically using a Thermo NanoDrop. Subsequently, cDNA synthesis was performed using the PrimeScript™ RT reagent kit (Takara, Dalian, China).

### 2.3. Selection of Reference Genes and Primer Formulation

The sequences of 11 candidate reference genes commonly used in insect studies, including *GAPDH*, *RPL32*, *RPL7A*, *RPS18*, *RPL13a*, *RPL18*, *Actin*, *RPS7*, *RPS3*, *VATB*, and *EF1A*, were obtained from the transcriptome data for *H. parallela* [20]. Primer design and evaluation were conducted using DNAMAN 8.0 software and Primer3 (v. 0.4.0) software (accessible at http://bioinfo.ut.ee/primer3/, accessed on 10 June 2022) following the principles for designing qRT-PCR primers.

### 2.4. Fluorescent Quantitative PCR Reaction Conditions

RT-qPCR was performed on the CFX384 Real-Time PCR Detection System (Bio-Rad, Hercules, CA, USA) using TB Green^®^ Premix Ex Taq™ II kit (Takara, Dalian, China). Primer sequences are detailed in Table 1. Each 10 μL reaction contained 5 μL of TB Green Premix, 0.4 μL of each primer, 0.8 μL of cDNA, and 3.4 μL of sterile water. The thermal cycling conditions included an initial denaturation step followed by 40 cycles of denaturation, annealing, and extension, and a final melting curve analysis. Melting curve and standard curve were conducted to ensure specificity and assess amplification efficiency. A standard curve was constructed using serial 10-fold dilutions of cDNA, which yielded the following five concentration gradients of cDNA templates: 10^0^ (500 ng/μL), 10^−1^, 10^−2^, 10^−3^, and 10^−4^. The amplification efficiency (E) value was then calculated for all primers as a percentage, as follows: E = (10^[−1/slope]^−1) × 100%.

### 2.5. Assessment of Reference Gene Stability

To assess the stability of candidate reference genes, this study employed a range of algorithms, including geNorm [1], NormFinder [28], BestKeeper [29], and the ΔCt method [30], providing a comprehensive evaluation. After this thorough assessment, the RefFinder [31] was employed to compare and rank the candidate genes, facilitating their prioritization.

### 2.6. Validation of Selected Reference Genes

The forkhead transcription factor *FoxL2* gene and the vitellogenin receptor (*VgR*) gene are associated with ovarian differentiation and egg development in insects. In this study, *FoxL2* and *VgR* were chosen as the target genes. To standardize the data, diverse internal reference genes were utilized and their stability was verified. The expression profiles of the target genes, *VgR* and *FoxL2*, were determined utilizing the 2^−ΔΔCt^ method. The SPSS Statistics 22 software package was then applied to evaluate the variance in expression levels of the genes of interest through an independent-samples *t*-test.

## 3. Results

### 3.1. Assessment of Primer Efficiency and Specificity in Amplification

Prior to assessing the suitability of reference genes, rigorous validation of the PCR amplification’s specificity and efficiency is essential. Each PCR amplicon was examined using a 1.5% agarose gel and displayed a single band that matched the anticipated length. Furthermore, the melting curves of PCR amplifications with each primer set exhibited a unique peak, demonstrating their specificity (Figure S1). All primer pairs, excluding the VATB primer, demonstrated high amplification efficiency (ranging from 105.82% to 114.35%), with regression coefficients exceeding the threshold of 0.990, indicating reliable qPCR results (Table 1, Figure S2).

### 3.2. Expression Profile of Reference Genes

**Figure 1:**
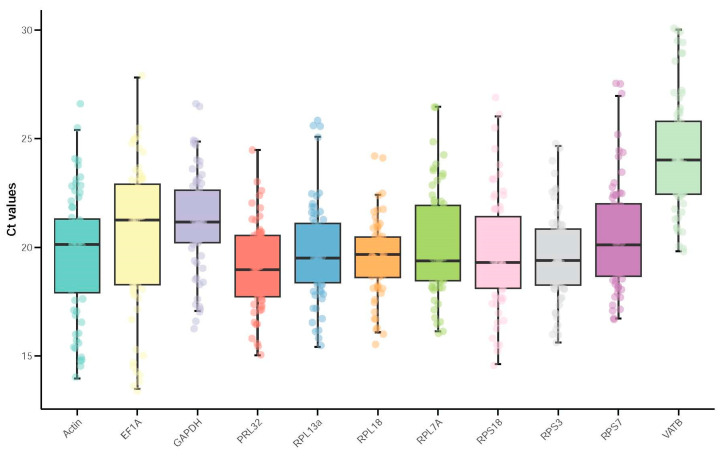
Reference Gene Expression under Different Treatments in *Holotrichia parallela*. In each box, the lower quartile (25th percentile)and upper quartile (75th percentile) are depicted. The whiskers mark the minimum and maximum values of the dataset. The horizontal line within each box marks the median value. Dots represent the reference gene expression for each sample.

The threshold-cycle (Ct) represents the transcript level of the mRNA. Under varying treatment conditions, the Ct values of the 11 candidate reference genes for *H. parallela* ranged from 13.48 to 30.01. The mean Ct values spanned a range, with *RPL32* exhibiting the lowest value at 19.05 and *VATB* having the highest value among the samples at 24.36 (Figure 1). Based on the coefficient of variation (CV), *RPL18* exhibited the highest stability (CV = 8.92) while *RPL32* displayed the greatest variability (CV = 15.94). (Table S2).

### 3.3. Identifying Reference Genes with Consistent Expression Stability

**Figure 2:**
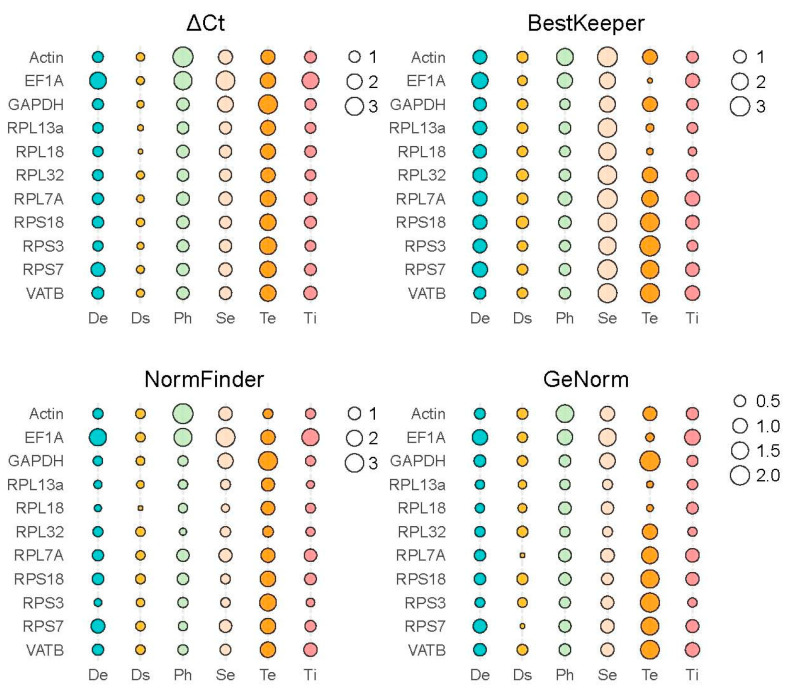
Stability of 11 candidate reference genes assessed using the ΔCt method, BestKeeper, NormFinder, and geNorm. The size of each bubble represents the reference gene stability value, with smaller bubbles indicating more stable genes. De: developmental stage, Ds: dsRNA treatment, Ph: photoperiod treatment, Se: sexes, Te: temperature, Ti: tissues.

The expression stability of 11 candidate internal reference genes in *H. parallela* under varying treatment conditions was assessed using the following four analysis methods: the ΔCt method, geNorm, NormFinder, and BestKeeper. RefFinder was used to comprehensively evaluate the expression stability of 11 candidate reference genes in *H. parallela* under different treatment conditions (Figure 2, Table S3).

**Figure 3:**
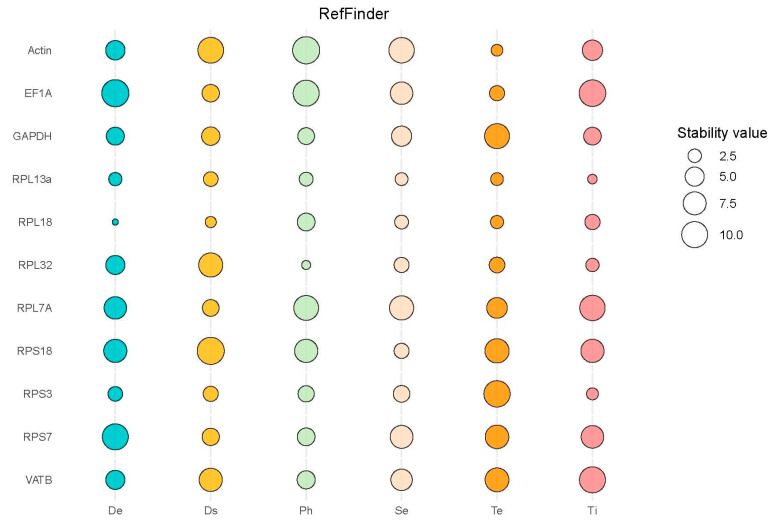
Expression stability of 11 reference genes of *Holotrichia parallela* under various treatment conditions analyzed using RefFinder. Bubble size represents reference gene stability, with smaller bubbles indicating greater stability. De: developmental stage, Ds: dsRNA treatment, Ph: photoperiod treatment, Se: sexes, Te: temperature, Ti: tissues.

For different developmental stages of *H. parallela*, the expression analysis of candidate reference genes was performed using the ΔCt method and NormFinder (Version dated 05/01-2015) software . It was found that *RPL18*, *RPS3*, and *RPL13a* showed the highest expression stability. The geNorm analysis revealed that *RPL18* and *RPL13a* were the most stable reference genes, while BestKeeper analysis indicated that the reference gene *VATB* was the most stable. All four analyses revealed that *RPS7* and *EF1A* were relatively unstable internal reference genes (Figure 2, Table S3). RefFinder analysis ranked the expression stability of candidate reference genes in *H. parallela* during development from highest to lowest, as follows: *RPL18*>*RPL13a*>*RPS3*>*GAPDH*>*RPL32*>*VATB*>*Actin*>*RPL7A*>*RPS18*>*RPS7*>*EF1A* (Figure 3, Table S3).

For female and male *H. parallela* tissues, analysis using the ΔCt method showed that *RPL13a* and *RPS18* exhibited the most stable expression. The geNorm software (Version 3.5) evaluation recognized *RPL32* and *RPL13a* as the top stable reference genes, whereas NormFinder pointed to *RPL18* as exhibiting the highest expression stability. All three analyses revealed that the expression of *GAPDH* and *EF1A* exhibited relatively poor stability. However, the BestKeeper software (Version 1) analysis suggested that *GAPDH* and *EF1A* showed the most stable expression while *RPL7A* and *Actin* exhibited poorer stability (Figure 2, Table S3). The RefFinder analysis ranked the expression stability of candidate reference genes in male and female adult *H. parallela* tissues from highest to lowest, as follows: *RPL13a*>*RPL18*>*RPL32*>*RPS18*>*RPS3*>*GAPDH*>*VATB*>*EF1A*>*RPS7*>*RPL7A*>*Actin* (Figure 3, Table S3).

For different tissues of *H. parallela*, the ΔCt analysis and NormFinder revealed that *RPL13a* exhibited the most stable expression among the candidate internal reference genes. The geNorm analysis identified *RPL32* and *RPS3* as the most stable reference genes. All three analyses indicated relatively poor stability in the expression of *VATB* and *EF1A*. According to the BestKeeper analysis,*RPL18* and *RPL13a* had the most stable expression, while *VATB* and *RPL7A* exhibited poorer stability (Figure 2, Table S3). The RefFinder analysis ranked the expression stability of candidate reference genes in different adult *H.parallela* tissues from highest to lowest, as follows: *RPL13a*>*RPS3*>*RPL32*>*RPL18*>*GAPDH*>*Actin*>*RPS7*>*RPS18*>*RPL7A*>*VATB*>*EF1A* (Figure 3, Table S3).

For different light exposure treatments, *RPL32* and *RPL13a* were identified as the most stable reference genes in adult *H. parallela* based on the ΔCt and geNorm analyses. Specifically, NormFinder favored *RPL32*, while BestKeeper suggested *GAPDH* as the preferred option. All four analyses demonstrated that *EF1A* and *Actin* had relatively poor expression stability (Figure 2, Table S3). RefFinder analysis ranked the expression stability of candidate reference genes in *H. parallela* under varying light exposure conditions from highest to lowest, as follows: *RPL32*>*RPL13a*>*RPS3*>*GAPDH*>*RPL18*>*RPS7*>*VATB* >*RPS18*>*RPL7A*>*EF1A*>*Actin* (Figure 3, Table S3).

For samples of third-instar *H. parallela* larvae under different temperature treatments, *Actin* and *RPL32* were identified as the most stable reference genes in adult *H. parallela*, based on the ΔCt and geNorm analyses. The geNorm analysis showed that *RPL13a* and *RPL18* were the most stable, while all three analyses demonstrated that *RPS3* and *GAPDH* were relatively unstable internal reference genes. The BestKeeper analysis identified *EF1A* and *RPL18* as the most stable, while *VATB* and *RPS3* exhibited poor stability (Figure 2, Table S3). The RefFinder analysis ranked the expression stability of candidate reference genes in third-instar *H. parallela* larvae samples under different temperature treatments from highest to lowest, as follows: *Actin*>*RPL13a*>*RPL18*>*EF1A*>*RPL32*>*RPL7A*>*RPS7*>*VATB* >*RPS18*>*GAPDH*>*RPS3* (Figure 3, Table S3).

For samples of *H. parallela* under RNAi feeding treatment, *RPL18* and *RPL13a* were identified as the most stable reference genes in adult *H. parallela* based on the ΔCt and geNorm analyses. While the geNorm analysis favored *RPL7A* and *RPS7*, the BestKeeper analysis identified *EF1A* as the most stable. All four analyses revealed that *RPS18* had relatively poor expression stability (Figure 2, Table S3). The RefFinder analysis ranked the expression stability of candidate reference genes in third-instar *H. parallela* larvae samples under RNAi feeding treatment from highest to lowest, as follows: *RPL18*>*RPL13a* >*RPS3*>*RPL7A*>*RPS7*>*EF1A*>*GAPDH*>*VATB*>*RPL32*>*Actin*>*RPS18* (Figure 3, Table S3).

### 3.4. Optimal Number of Reference Genes

**Figure 4:**
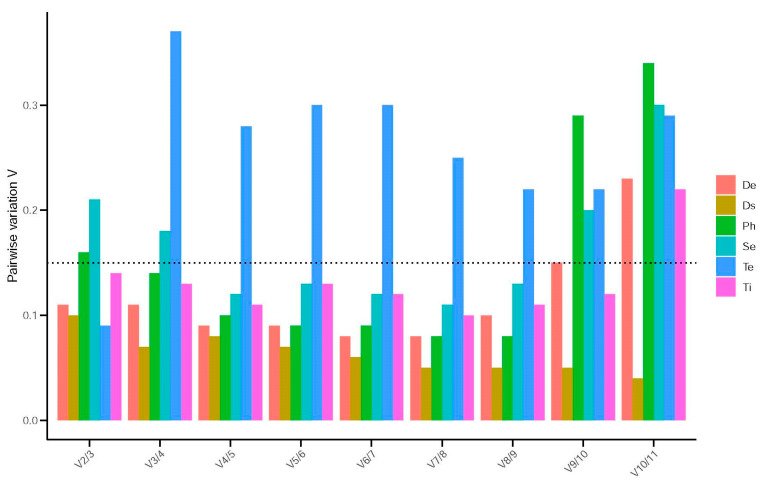
geNorm analysis of 11 reference genes in *Holotrichia parallela* determined the optimal number for accurate normalization. Pairwise variation (Vn/Vn+1) values indicate the stability of reference gene combinations. The dotted line represents the recommended threshold (0.15), below which additional reference genesare not necessary. De: developmental stage, Ds: dsRNA treatment, Ph: photoperiod treatment, Se: sexes, Te: temperature, Ti: tissues.

Pairwise variation (V) values, which indicate the stability of reference genes, were computed using thegeNorm software to ascertain the optimal number of reference genes required for data normalization under different experimental conditions. The results of the analysis showed that for different developmental stages and adult tissues, V2/3 to V9/10 were all less than 0.15. For third-instar larvae under varying temperature conditions, V2/3 was less than 0.15 and V3/4 to V10/11 were all greater than 0.15. Additionally, for adult *H. parallela* under RNAi feeding treatment, V2/3 to V10/11 were all less than 0.15. This suggests that two candidate reference genes are sufficient for data normalization in different developmental stages of *H. parallela*, adult *H. parallela* tissues, third-instar *H. parallela* larvae under varying temperature conditions, and adult *H. parallela* under RNAi feeding treatment. For different tissues of adult male and female *H. parallela*, V4/5 to V8/9 were all less than 0.15 while V2/3 to V3/4 and V9/10 to V10/11 were all greater than 0.15. Under different light conditions, V3/4 to V8/9 were all less than 0.15 while V2/3 and V9/10 to V10/11 were all greater than 0.15. This indicates that three candidate reference genes should be used for data standardization in the different tissues of male and female adult *H. parallela* as well as in adult *H. parallela* under varying light conditions (Figure 4).

### 3.5. The Influence of Housekeeping Genes on Quantitative PCR Data Interpretation

The stability analysis and pairwise variation (V) calculations of comprehensive candidate reference genes performed under the *H. parallela* RNAi feeding treatment indicate that utilizing two highly stable reference genes is optimal for normalization. Consequently, in this research, the pairing of the most stable genes *RPL18* and *RPL13a*, in addition to the least stable gene, *RPS18*, were chosen as reference genes. These genes were employed to analyze the expression levels of the vitellogenin receptor (*VgR*) gene and the forkhead box L2 (*FoxL2*) gene, ultimately validating the stability of the selected reference genes.

**Figure 5:**
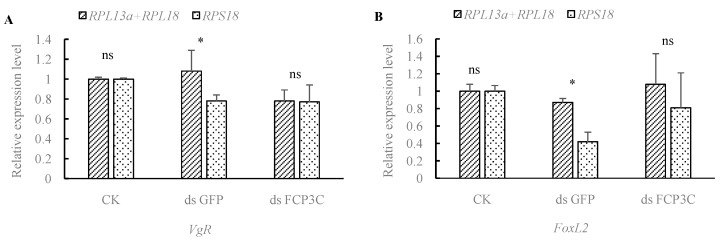
Expression of the target genes *VgR* (**A**) and *FoxL2* (**B**) in *Holotrichia parallela* treated with RNA interference (RNAi) using different reference genes. *: a statistically significant difference with *p* < 0.05; ns: not significant.

The follicular cell protein (*Fcp3C*) gene is related to insect reproduction and oviposition. After RNAi, *Fcp3C* affects the expression of the *VgR* and *FoxL2* genes. When using the highly stable gene combination of *RPL18* and *RPL13a* as reference genes, the expression level of the target gene *VgR* was relatively lower in dsFcp3C samples. Utilizing the least stable gene, *RPS18*, as a reference gene, the expression level of the target gene *VgR* was relatively lower in both the dsGFP and dsFcp3C samples, with no significant difference between them (Figure 5A). Regarding the expression level of the target gene *FoxL2*, when using the highly stable gene combination of *RPL18* and *RPL13a* as reference genes, the expression level in dsFcp3C samples was higher than in the control (ddH_2_O) and dsGFP samples. There was no significant difference in expression level between the control (ddH_2_O) and dsGFP samples. However, when using *RPS18* (the least stable gene) as a reference gene, the expression level of the target gene *FoxL2* was higher in the control (ddH_2_O) sample, whereas it was lower in both the dsGFP and dsFcp3C samples, with the lowest expression level being observed in the dsGFP sample (Figure 5B). Based on the validation results discussed above, using different reference genes for normalizing target genes will indeed yield different experimental results.

## 4. Discussion

qPCR, which is usually used to determine gene expression levels, is a widely recognized, established method that serves as a gold standard in molecular biology research [32,33,34]. Normalization is essential in qRT-PCR analysis for addressing variations that occur within and between reaction cycles. These variations can lead to inconsistencies, including variations in the quantity of the initial sample material, differences in RNA integrity, discrepancies in the loading amounts of cDNA among samples, and deviations in reverse transcription efficiency [35,36,37]. Thus, the observed reference gene variability encapsulates both the intrinsic variability of the gene itself and the technical errors incurred during the process [36]. Controlling all steps leading up to the PCR measurement is crucial, and selecting an appropriate reference gene is vital for achieving precision across varying experimental setups.

Transcriptomic datasets for *H. parallela* have been made available to facilitate research into its developmental processes, chemosensory genes, odorant-degrading enzymes, and immune responses [19,20,38,39]. Previous qRT-PCR studies on *H. parallela* generally utilized conventional insect reference genes, notably *Actin* and *GAPDH* [21,38,40,41]. In addition to the highest stability of *Actin* under different temperature conditions, the present study found that *Actin* and *GAPDH* exhibited particularly high variable instability values. Therefore, neither *Actin* nor *GAPDH* was the most stable reference gene in most cases. The expression of *Actin* was less stable in several insects, including *Tribolium castaneum*, *H. oblita, Coleomegilla maculata*, *Hippodamia convergen* and *Hippodamia variegata*, all belonging to the order Coleoptera [27,42,43,44,45].

The present study identified unique gene stability profiles in *H. parallela*, aligning with previous research findings [27] that highlighted the variability of gene stability under various experimental conditions in *Holotrichia oblita*. In the present study on *H. parallela*, *RPL18* and *RPL13a* were the most stable across the developmental stages and RNAi conditions. This suggests that these two genes can serve as reliable reference gene selections under these experimental conditions. Furthermore, *RPL13a*, *RPL18*, and *RPL32* displayed high stability in both sexes, indicating their appropriateness as reference genes for sex-specific gene expression analyses in *H. parallela*. Under varying photoperiods, *RPL32*, *RPL13a*, and *RPS3* exhibited the greatest stability, while *Actin* and *RPL13a* were the most stable across different temperatures.

In contrast, previous studies on *H. oblita* have reported that *RPL13a* and *RPL18* are the optimal reference genes for investigating different tissues, developmental stages, and temperature. This is in line with the present findings in *H. parallela*. The consistency in the stability of *RPL13a* and *RPL18* across species suggests that these genes may serve as general reference markers for gene expression analysis in closely related insects, particularly within the *Holotrichia* genus. However, for photoperiod treatments in *H. oblita*, *RPL13a* and *RPS3* were found to be the most stable genes, while *RPS18* and *RPL18* were most suitable for sex-specific gene expression studies. Among the selected genes, including *GAPDH*, *Actin*, and 18s rRNAs, *GAPDH* was selected as a standardized reference gene in various tissues of *H. parallela* [38]. However, when compared to the other genes analyzed in the current study, *RPL18* and *RPL13a* exhibited the greatest stability, while *GAPDH* ranked sixth in stability among the 11 genes tested. Similarly, *Actin* consistently ranked below the median in stability assessments, except when it exhibited the highest stability under different temperature treatments, which supports findings observed in other species [43,45,46,47]. In a previous work, *GAPDH* and *RPL13a* were considered the best reference genes for various tissues and the developmental stages of *Anomala corpulenta*, respectively [48].

Extensive studies across insect species have confirmed that ribosomal proteins are the most reliable reference genes, excelling in expression consistency and robustness compared to other candidates [22]. Consistently, the results of the current analysis ranked ribosomal protein genes among the three most stable reference gene options across diverse experimental settings. In the field of gene expression studies in insects, the ribosomal protein gene, including *RPL18*, *RPS23, PRL13*, and *RPL32*, has emerged as the preferred choice for reference genes, demonstrating superior suitability based on recent comprehensive evaluations [44,49,50,51,52]. Furthermore, *RPL18*, *RPS3*, and *RPL13a* can be considered as proper reference genes for normalizing RT-qPCR data, especially when choosing reference genes from other Coleoptera insects that have not been extensively studied. Nevertheless, the differences observed in the stability of other genes under specific conditions highlight the crucial role of choosing suitable reference genes based on the experimental plan and species under investigation.

In summary, this study offers significant insights into the identification of appropriate reference genes for quantitative gene expression analysis in *H. parallela,* especially within the context of RNAi treatments. This will guarantee precise and dependable analysis of gene expression for similar gene types in this and other closely related insect species and facilitate the more effective identification of target genes for pest control.

## Figures and Tables

**Table 1:** The primer sequences for the candidate reference genes and the two target genes utilized in this study.

Gene	Primer Sequences (5′-3′, F/R)	Efficieny (E) (%)	Regression Coefficient (R^2^)
GAPDH	AGTCGCCGTAAATGATCCCT	110.68	0.9971
	CGTCGACTGTGCCATTGAAT		
RPL32	GCAAAAACCCGTCATATGCT	110.17	0.998
	TGATACGCCATGTGCAATTT		
RPL7A	TGCAAATACAATCCGAGCTG	107.71	0.9916
	GGCAGGAAGAGCACAAGTTC		
RPS18	CGTGCTGGAGAATGTTCTGA	105.82	0.9972
	GCTGGCTATATTTGCCATCAA		
RPL13a	GGCGTACCTCCACCTTATGA	106.76	0.9978
	CAGAATTTGCGACCAGGTTT		
RPL18	CATTACGCTCACCAACAGGA	111.19	0.998
	CTGGAGCAGGACCAAAGTGT		
Actin	TGTCACTGTATGCCTCTGGT	109.67	0.9978
	TACCAGCCAAATCCAAACGC		
RPS7	CGCGAGCTTGAGAAGAAGTT	109.18	0.9994
	AGAACGTGGACGCTTCTGTT		
RPS3	ATCCACTCAGGTGACCCTTG	114.35	0.9956
	AACGGCCTCTTAGGTCCAAT		
VATB	GGTCTACCGCACAACGAAAT	126.92	0.9933
	ACCTAGCGGTTTCCATGTTG		
EF1A	GCCAGAAGCTGTACCTGGAG	106.76	0.9994
	TGTCACCGGCTACATAACCA		
Target gene			
VgR	TGGCGAAGACGAGAAAAACT	-	-
	TCGTCCGACAAATCGTAACA		
FoxL 2	CAGCAGCCTATACGCAACAA	-	-
	AGGAGGCCAATAAGCTGGAT		

## Data Availability

The original contributions presented in the study are included in the article and Supplementary Materials; further inquiries can be directed to the corresponding author.

## Supplementary Materials

The following supporting information can be downloaded at: https://www.mdpi.com/article/10.3390/insects15090661/s1, Figure S1: Specificity of primer pairs; Figure S2: Amplification efficiency curves; Table S1: Standard curve equations, slopes, and amplification efficiencies of the 11 candidate internal reference genes; Table S2: Ct values and coefficients of variation (CV) for the 11 candidate internal reference genes Table S3: The stability of the 11 candidate reference genes evaluated by ΔCt method, BestKeeper, NormFinder, and geNorm.

## References

[1] Vandesompele J., De Preter K., Pattyn F., Poppe B., Van Roy N., De Paepe A., Speleman F. (2002). Accurate normalization of real-time quantitative RT-PCR data by geometric averaging of multiple internal control genes. Genome Biol..

[2] Thellin O., Zorzi W., Lakaye B., De Borman B., Coumans B., Hennen G., Grisar T., Igout A., Heinen E. (1999). Housekeeping genes as internal standards: Use and limits. J. Biotechnol..

[3] Gutierrez L., Mauriat M., Guénin S., Pelloux J., Lefebvre J.F., Louvet R., Rusterucci C., Moritz T., Guerineau F., Bellini C. (2008). The lack of a systematic validation of reference genes: A serious pitfall undervalued in reverse transcription-polymerase chain reaction (RT-PCR) analysis in plants. Plant Biotechnol. J..

[4] Wei H., Qiao H., Liu S., Yuan X., Xu C. (2023). Transcriptome-based selection and validation of reference genes for gene expression in Goji Fruit Fly (*Neoceratitis asiatica* Becker) under developmental stages and five abiotic stresses. Int. J. Mol. Sci..

[5] Dong X.M., Zhang W., Zhang S.B. (2022). Selection and validation of reference genes for quantitative real-time PCR analysis of development and tissue-dependent flower color formation in *Cymbidium lowianum*. Int. J. Mol. Sci..

[6] Shi C., Yang F., Zhu X., Du E., Yang Y., Wang S., Wu Q., Zhang Y. (2016). Evaluation of housekeeping genes for quantitative real-time PCR analysis of *Bradysia odoriphaga* (Diptera: Sciaridae). Int. J. Mol. Sci..

[7] Hruz T., Wyss M., Docquier M., Pfaffl M.W., Masanetz S., Borghi L., Verbrugghe P., Kalaydjieva L., Bleuler S., Laule O. (2011). RefGenes: Identification of reliable and condition specific reference genes for RT-qPCR data normalization. BMC Genom..

[8] Zhang M., Cui Z., Zhang N., Xie G., Wang W., Chen L. (2021). Electrophysiological and behavioral responses of *Holotrichia parallela* to volatiles from peanut. Insects.

[9] Li E.T., Zhang S., Li K.B., Nyamwasaa I., Li J.Q., Li X.F., Qin J.H., Yin J. (2021). Efficacy of entomopathogenic nematode and *Bacillus thuringiensis* combinations against *Holotrichia parallela* (Coleoptera: Scarabaeidae) larvae. Biol. Control.

[10] Liu Q., Li J., Xu X., Sun C., Kang Y., Zhou H., Hu D., Ma J., Li S. (2007). The preliminary study on grub control with *Rhabditis* (*Oscheius*) spp in peanut fields. Acta Agric. Boreali Sin..

[11] Toepfer S., Li H., Pak S.G., Son K.M., Ryang Y.S., Kang S.I., Han R., Holmes K. (2014). Soil insect pests of cold temperate zones of East Asia, including DPR Korea: A review. J. Pest Sci..

[12] Zhang M., Yin J., Li K., Cao Y. (2014). Research progress on the occurrences of white grub and its control. China Plant Prot..

[13] Pei G., Ma S., Liu J., Liu B. (2010). Effects of different cultivation patterns on grubs occurrence and yield in soybean fields. Soybean Bull.

[14] Liu S., Li K., Yin J., Cao Y. (2008). Review of the researches on biological control of grubs. Chin. J. Biol. Control.

[15] Koo J., Palli S.R. (2024). Recent advances in understanding of the mechanisms of RNA interference in insects. Insect Mol. Biol..

[16] Bargmann C.I. (2001). High-throughput reverse genetics: RNAi screens in *Caenorhabditis elegans*. Genome Biol..

[17] De Schutter K., Taning C.N.T., Van Daele L., Van Damme E.J., Dubruel P., Smagghe G. (2022). RNAi-based biocontrol products: Market status, regulatory aspects, and risk assessment. Front. Insect Sci..

[18] Li E.T., Wu H.J., Qin J.H., Luo J., Li K.B., Cao Y.Z., Zhang S., Peng Y., Yin J. (2023). Involvement of *Holotrichia parallela* odorant-binding protein 3 in the localization of oviposition sites. Int. J. Biol. Macromol..

[19] Li E., Qin J., Feng H., Li J., Li X., Nyamwasa I., Cao Y., Ruan W., Li K., Yin J. (2021). Immune-related genes of the larval *Holotrichia parallela* in response to entomopathogenic nematodes *Heterorhabditis beicherriana* LF. BMC Genom..

[20] Gong Z., Zhang J., Li Y., Li H., Zhang Z., Qin Y., Jiang Y., Duan Y., Li T., Miao J. (2023). Identification of potential gene targets for suppressing oviposition in *Holotrichia parallela* using comparative transcriptome analysis. Int. J. Mol. Sci..

[21] Zhao D., Liu Z.R., Wu H., Fu C.R., Li Y.Z., Lu X.J., Guo W. (2021). RNA interference-mediated functional characterization of Group I chitin deacetylases in *Holotrichia parallela* Motschulsky. Pestic. Biochem. Physiol..

[22] Shakeel M., Rodriguez A., Tahir U.B., Jin F. (2018). Gene expression studies of reference genes for quantitative real-time PCR: An overview in insects. Biotechnol. Lett..

[23] Shi C.H., Hu J.R., Zhang Y.J. (2015). Research progress on reference genes of insect for quantitative real-time reverse transcription PCR (RT-qPCR). Univers. J. Agric. Res..

[24] Lü J., Yang C., Zhang Y., Pan H. (2018). Selection of reference genes for the normalization of RT-qPCR data in gene expression studies in insects: A systematic review. Front. Physiol..

[25] Zhou L., Ju Q., Qu M., Ye M., Wang L., Zhao Z., Du L. (2008). The research on artificial rearing of Holotrichia parallela and its susceptive to insecticides. J. Peanut Sci..

[26] Xie M., Zhong Y., Lin L., Zhang G., Su W., Ni W., Qu M., Chen H. (2022). Transcriptome analysis of *Holotrichia oblita* reveals differentially expressed unigenes related to reproduction and development under different photoperiods. Comp. Biochem. Physiol. D.

[27] Xie M., Zhong Y., Lin L., Zhang G., Su W., Ni W., Qu M., Chen H. (2020). Evaluation of reference genes for quantitative real-time PCR normalization in the scarab beetle *Holotrichia oblita*. PLoS ONE.

[28] Andersen C.L., Jensen J.L., Ørntoft T.F. (2004). Normalization of real-time quantitative reverse transcription-PCR data: A model-based variance estimation approach to identify genes suited for normalization, applied to bladder and colon cancer data sets. Cancer Res..

[29] Pfaffl M.W., Tichopad A., Prgomet C., Neuvians T.P. (2004). Determination of stable housekeeping genes, differentially regulated target genes and sample integrity: BestKeeper–Excel-based tool using pair-wise correlations. Biotechnol. Lett..

[30] Silver N., Best S., Jiang J., Thein S.L. (2006). Selection of housekeeping genes for gene expression studies in human reticulocytes using real-time PCR. BMC Mol. Biol..

[31] Xie F., Wang J., Zhang B. (2023). RefFinder: A web-based tool for comprehensively analyzing and identifying reference genes. Funct. Integr. Genom..

[32] Bustin S.A., Beaulieu J.-F., Huggett J., Jaggi R., Kibenge F.S., Olsvik P.A., Penning L.C., Toegel S. (2010). MIQE precis: Practical implementation of minimum standard guidelines for fluorescence-based quantitative real-time PCR experiments. BMC Mol. Biol..

[33] Radonić A., Thulke S., Mackay I.M., Landt O., Siegert W., Nitsche A. (2004). Guideline to reference gene selection for quantitative real-time PCR. Biochem. Biophys. Res. Commun..

[34] Kozera B., Rapacz M. (2013). Reference genes in real-time PCR. J. Appl. Genet..

[35] Chervoneva I., Li Y., Schulz S., Croker S., Wilson C., Waldman S.A., Hyslop T. (2010). Selection of optimal reference genes for normalization in quantitative RT-PCR. BMC Bioinform..

[36] Huggett J., Dheda K., Bustin S., Zumla A. (2005). Real-time RT-PCR normalisation; strategies and considerations. Genes Immun..

[37] Wong M.L., Medrano J.F. (2005). Real-time PCR for mRNA quantitation. Biotechniques.

[38] Yi J., Wang S., Wang Z., Wang X., Li G., Zhang X., Pan Y., Zhao S., Zhang J., Zhou J.J. (2021). Identification of candidate carboxylesterases associated with odorant degradation in *Holotrichia parallela* antennae based on transcriptome analysis. Front. Physiol..

[39] Shu C., Tan S., Yin J., Soberón M., Bravo A., Liu C., Geng L., Song F., Li K., Zhang J. (2015). Assembling of *Holotrichia parallela* (dark black chafer) midgut tissue transcriptome and identification of midgut proteins that bind to Cry8Ea toxin from *Bacillus thuringiensis*. Appl. Microbiol. Biotechnol..

[40] Zhao D., Liu X., Liu Z., Lu X., Guo W. (2022). Identification and functional analysis of two potential RNAi targets for chitin degradation in *Holotrichia parallela* Motschulsky (Insecta Coleoptera). Pestic. Biochem. Physiol..

[41] Zhao D., Guo W., Li S., Li R., Xu D., Lu X. (2014). Identification of a new peritrophic membrane protein from larval *Holotrichia parallela* (Coleoptera: Motschulsky). Molecules.

[42] Lord J.C., Hartzer K., Toutges M., Oppert B. (2010). Evaluation of quantitative PCR reference genes for gene expression studies in *Tribolium castaneum* after fungal challenge. J. Microbiol. Meth..

[43] Pan H., Yang X., Siegfried B.D., Zhou X. (2015). A comprehensive selection of reference genes for RT-qPCR analysis in a predatory lady beetle, *Hippodamia convergens* (Coleoptera: Coccinellidae). PLoS ONE.

[44] Xie J., Liu T., Khashaveh A., Yi C., Liu X., Zhang Y. (2021). Identification and evaluation of suitable reference genes for RT-qPCR analysis in *Hippodamia variegata* (Coleoptera: Coccinellidae) under different biotic and abiotic conditions. Front. Physiol..

[45] Yang C., Pan H., Noland J.E., Zhang D., Zhang Z., Liu Y., Zhou X. (2015). Selection of reference genes for RT-qPCR analysis in a predatory biological control agent, *Coleomegilla maculata* (Coleoptera: Coccinellidae). Sci. Rep..

[46] Yang C., Preisser E.L., Zhang H., Liu Y., Dai L., Pan H., Zhou X. (2016). Selection of reference genes for RT-qPCR analysis in *Coccinellas eptempunctata* to assess un-intended effects of RNAi transgenic plants. Front. Plant Sci..

[47] Yang X., Pan H., Yuan L., Zhou X. (2018). Reference gene selection for RT-qPCR analysis in *Harmonia axyridis*, a global invasive lady beetle. Sci. Rep..

[48] Chen H., Qu M., Ali F., Lin L., Xie M., Zhang G., Su W. (2019). Expression analysis of odorant-binding protein genes and chemosensory protein genes in *Anomala corpulenta* Motschulsky (Coleoptera: Scarabaeidae). J. Kans. Entomol. Soc..

[49] Wang L., Yang C., Liu Q., Zhang X., Mei X., Zhang T., Ning J. (2024). Validation and evaluation of reference genes for quantitative real-time PCR analysis in *Mythimna loreyi* (Lepidoptera: Noctuidae). Insects.

[50] Wang L., Liu Q., Guo P., Gao Z., Chen D., Zhang T., Ning J. (2023). Evaluation of reference genes for quantitative real-time PCR analysis in the Bean Bug, *Riptortuspedestris* (Hemiptera: Alydidae). Insects.

[51] Xue D., Chen T., Wu Y. (2024). Stability evaluation of candidate reference genes for real-time qPCR normalization in *Rhyzopertha dominica* (Coleoptera: Bostrycidae). J. Econ. Entomol..

[52] Shen C.H., Tang M., Li X.-F., Zhu L., Li W., Deng P., Zhai Q., Wu G., Yan X.H. (2024). Evaluation of reference genes for quantitative expression analysis in *Mylabris sibirica* (Coleoptera, Meloidae). Front. Physiol..

