# Supplementary material for: Comprehensive Screening and Validation of Stable Internal Reference Genes for Accurate qRT-PCR Analysis in Holotrichia parallela under Diverse Biological Conditions and Environmental Stresses
Source: Insects. 2024 Aug 30;15(9):661. doi: 10.3390/insects15090661 (PMC11432719; doi:10.3390/insects15090661)
Supplement: Supplementary file 1 [file insects-15-00661-s001.zip › Supplementary files/Supplementary_Material_Figures.pdf]

insects

MDPI

Article

Comprehensive Screening and Validation of Stable Internal Reference Genes for Accurate qRT-PCR Analysis in *Holotrichia parallela* under Diverse Biological Conditions and Environmental Stresses

Zhongjun Gong [1], Jing Zhang [1], Qi Chen [2], Huiling Li [1], Ziqi Zhang [3], Yun Duan [1], Yueli Jiang [1], Tong Li [1], Jin Miao [1] and Yuqing Wu [1*]

1 Institute of Plant Protection, Henan Academy of Agricultural Sciences, Key Laboratory of Crop Pest Control of Henan Province, Key Laboratory of Crop Integrated Pest Management of the Southern of North China, Ministry of Agriculture of the People's Republic of China, Zhengzhou 450002, People's Republic of China; gongzj_2@hotmail.com (G.Z.); xiamu1231@126.com (Z.J.); lihuiling05@163.com (L.H.); duanyunhao@163.com (D.Y.); yueli006@126.com (J.Y.); tongli84@hotmail.com (L.T.); miaojin1977@163.com (M.J.)

2 Luohe Academy of Agricultural Sciences, Luohe 462300, People's Republic of China; chenqi9992@sina.com (C.Q.)

3 Institute of Plant Protection, Luoyang Academy of Agricultural and Forestry Sciences, Luoyang 471027, People's Republic of China; lynkyzzq@126.com (Z.Z.)

* Correspondence: yuqingwu36@hotmail.com (W.Y.)

Citation: Gong, Z.; Zhang, J.; Chen, Q.; Li, H.; Zhang, Z.; Duan, Y.; Jiang, Y.; Li, T.; Miao, J.; Wu, Y. Comprehensive Screening and Validation of Stable Internal Reference Genes for Accurate qRT-PCR Analysis in *Holotrichia parallela* under Diverse Biological Conditions and Environmental Stresses. *Insects* **2024**, *15*, 661. https://doi.org/10.3390/insects15090661

Academic Editor: Vojislava Grbic

Received: date
Revised: date
Accepted: date
Published: 30 August 2024

Copyright: © 2024 by the authors. Submitted for possible open access publication under the terms and conditions of the Creative Commons Attribution (CC BY) license (https://creativecommons.org/licenses/by/4.0/).

Supplementary Figures

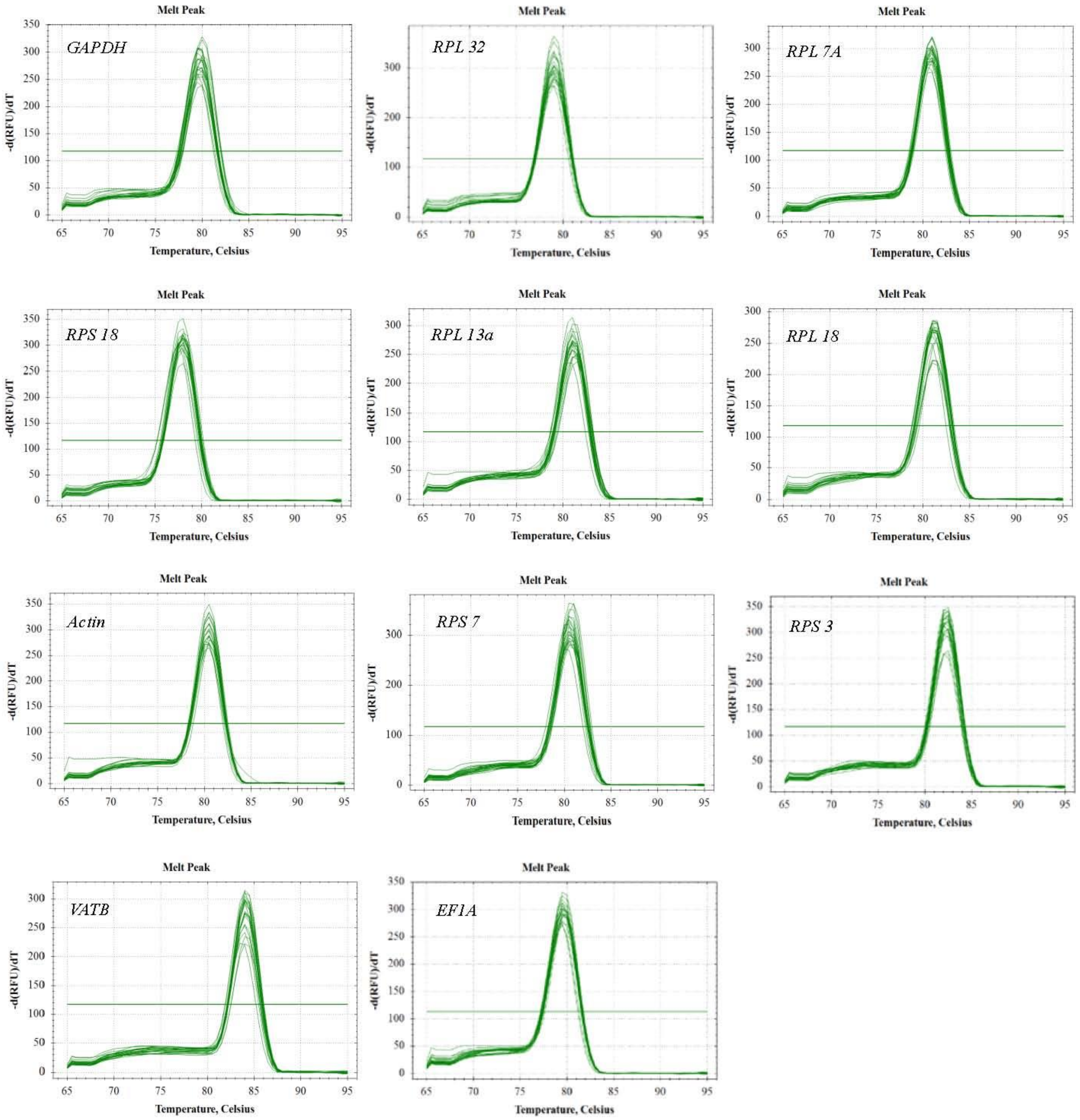

Figure S1. Specificity of primer pairs for qPCR amplification in *Holotrichia parallela*. The melt peaks of primers for qPCR amplification of 11 candidate reference genes (including *GAPDH*、*RPL32*、*RPL7A*、*RPS18*、*RPL13a*、*RPL18*、*Actin*、*RPS7*、*RPS3*、*VATB* and *EF1A*).

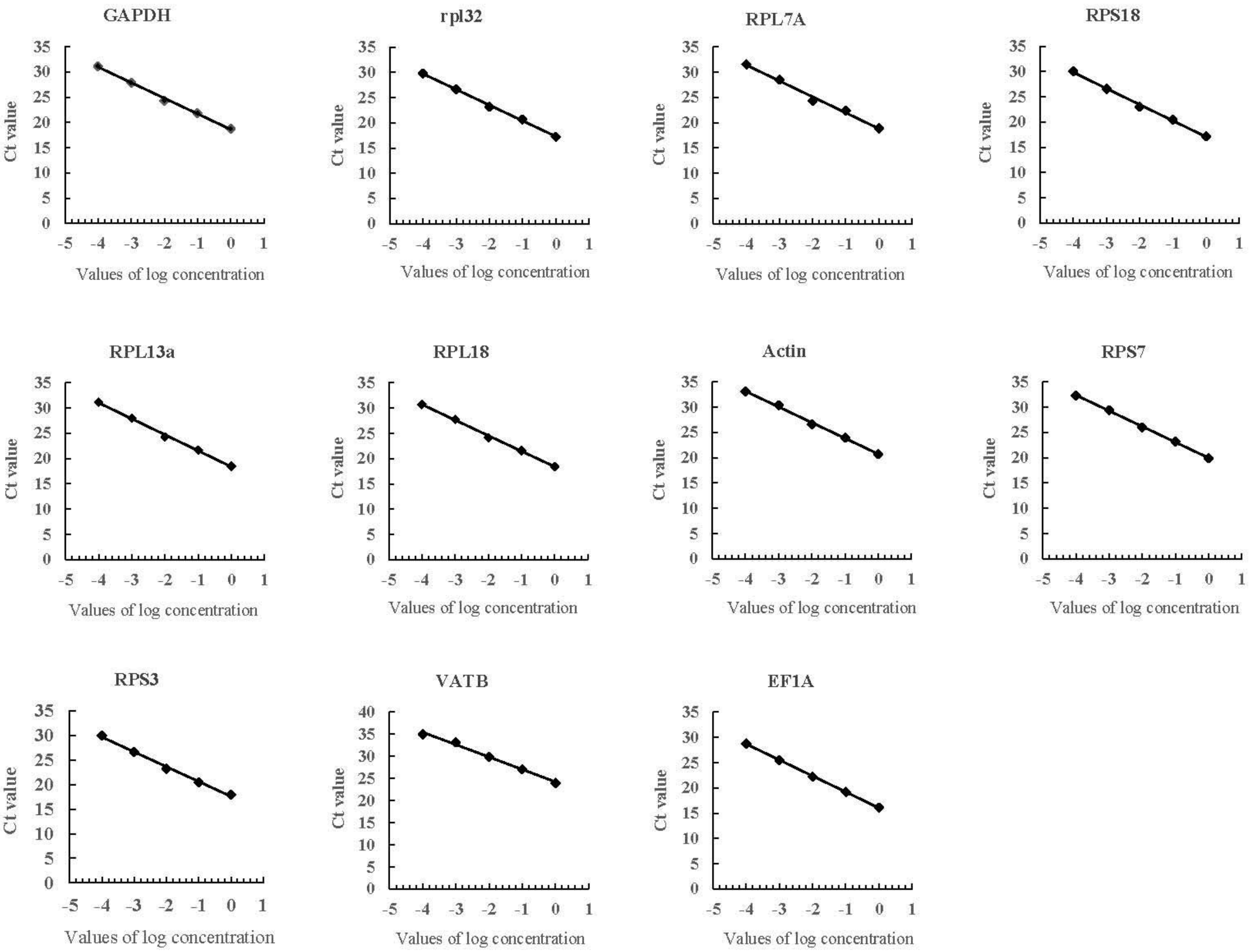

Figure S2. Amplification efficiency curves. Amplification efficiency curves of 11 known reference gene. The corresponding efficiency value (E), slope (K), and coefficient of determination of the fitted curve (R2) are shown per candidate reference gene (Table S1).
